# Neoadjuvant Chemoradiotherapy for Stage III Non-Small Cell Lung Cancer

**DOI:** 10.3389/fonc.2017.00281

**Published:** 2017-12-04

**Authors:** David J. Sher

**Affiliations:** ^1^Department of Radiation Oncology, Division of Outcomes and Health Services Research, UT Southwestern Medical Center, Dallas, TX, United States

**Keywords:** lung cancer, radiation therapy, neoadjuvant therapy, combined modality therapy, stage III non-small cell lung cancer

## Abstract

The local management of stage III non-small cell lung cancer is controversial. Although definitive chemoradiotherapy (CRT) is considered a standard-of-care in the curative management of the disease, inadequate local control outcomes have led to various treatment strategies that incorporate surgical resection. Surgery alone has long been recognized as insufficient for this stage, and thus neoadjuvant strategies have been developed to treat micrometastatic disease and increase the probability of a complete resection. The optimal induction strategy has not yet been defined, however, with arguments favoring either preoperative chemotherapy or CRT. In this article, the data supporting the use of neoadjuvant CRT and the randomized literature comparing the two approaches will be reviewed. The article will conclude with summary comparisons of these induction paradigms.

## Introduction

Although it is well-known that the successful treatment of stage III non-small cell lung cancer (NSCLC) is compromised by a high risk of micrometastatic disease, obtaining locoregional control has also long bedeviled local therapists. In the classic RTOG 73-01 study of radiation dose escalation in NSCLC, Perez et al. showed that the ultimate intrathoracic failure risks for squamous cell carcinoma and adenocarcinoma were 80 and 65%, respectively (1).

Additional non-invasive efforts to improve locoregional control first centered on altered fractionation approaches, and while there were some modest successes (2), none were paradigm shifting. The most important therapeutic change in the management of the disease arose from a series of landmark trials of chemotherapy. First, sequential chemotherapy and radiotherapy (RT) were shown to improve overall survival over RT alone (3), and then randomized trials of concurrent chemoradiotherapy (CRT) confirmed that concomitant treatment was clearly superior to single modality radiation treatment (4). The next generation of randomized studies showed that concurrent was superior to sequential delivery of chemotherapy, with the mode of improvement through superior locoregional control (5).

Yet despite this elegant progression of clinical investigation, definitive RT-based regimens still resulted in inadequate thoracic control rates. For example, the concurrent CRT arm of the RTOG 9410 trial, which helped to establish definitive CRT as a standard-of-care, still resulted in a crude thoracic failure risk of 45% (6). A more modern study of definitive CRT using the now favored carboplatin–paclitaxel regimen with 66 Gy resulted in a crude local failure risk of 36% (7). After multiple retrospective studies of radiation dose escalation, the definitive RTOG 0617 study randomized patients between 60 and 74 Gy of CRT, finding no difference in locoregional control or survival between the arms (8). Despite modern RT planning and near uniform PET-CT staging, the 2-year local failure risk was 30.7 and 38.6% for the 60 and 74 Gy arms, respectively. Given these humbling results, there have been longstanding efforts to integrate surgical resection into the curative paradigm of operable patients. The underlying concept is that surgical extirpation of potentially radioresistant disease would provide improved thoracic control that may translate into an overall survival benefit. In this article, the key prospective data that motivate treatment with preoperative CRT will be reviewed. Studies of preoperative chemotherapy versus upfront surgery will not be the subject of this review.

## SWOG 8805

The viability of preoperative CRT was shown in SWOG 8805, which was a multi-institutional phase II trial of induction CRT followed by anatomic resection (9). In this study, 126 patients with either N2 or N3 nodal disease and/or T4 primary lesions were treated with induction RT to 45 Gy with two concurrent cycles of etoposide–cisplatin. Patients with a complete resection and negative mediastinum were subsequently observed, and the remaining patients were treated with two additional cycles and consolidation RT to 59.4 Gy. Four patients experienced an early death (two treatment related), and 10 patients experienced progression of disease; 4 additional patients were ineligible for surgery.

Eleven percent of the remaining cohort had unresectable disease at thoracotomy. A pathologic complete response (pCR) was seen in 21% of resected patients, and 56% of patients with initial mediastinal nodal assessment experienced clearance of disease. Out of the entire initial cohort of 126 patients, there were a total of 25 first locoregional progressions (including synchronous metastases), resulting in a crude failure risk of 20%. The 3-year OS for patients with N2 disease at diagnosis was only 24%. However, among all patients with pathologically proven mediastinal adenopathy at diagnosis, the 3-year survival in patients with mediastinal nodal pCR versus not was 41 vs. 11% (*p* = 0.003), highlighting a consistent theme through the induction literature; namely, that patients with mediastinal clearance experience dramatically improved survival in comparison to those who do not.

The toxicity of trimodality therapy was not trivial. A total of 49 and 13% of patients experienced a grade 3 or grade 4 toxicity, respectively. Out of the 32 non-cancer related deaths, 13 were attributed to treatment, 8 of which were in the postoperative period. Six of these deaths were in patients who underwent a pneumonectomy, some of the initial data showing that the physiologic stress of post-CRT pneumonectomy may be profound.

## Alternative Dose-Fractionation Regimens

Because mediastinal pCR rates appear so closely linked to outcome, attempts have been made to increase mediastinal clearance through radiation dose intensification. For example, in a large phase II trial for patients with stage III NSCLC, investigators in Germany delivered four cycles of induction chemotherapy followed by 45 Gy in 3 weeks (1.5 Gy twice per day, BID) with concurrent carboplatin–paclitaxel, with surgery after radiation therapy (10). As opposed to most studies of trimodality therapy, this study cohort did not mandate operability at diagnosis. Of the 84 patients (out of 120) who were ultimately resectable, 58 (48% of the entire cohort) were completely resectable. The 30-day mortality was only 3%, but it was 11% (4 deaths) among the 36 patients who underwent pneumonectomy. The 5-year overall survival for all patients was 21.7% at 5 years, with the outcomes improving to 32.3% for individuals with stage IIIA disease; this latter number is quite favorable in comparison to most series for this stage. Patients with nodal pCR (*n* = 30, 25% of entire cohort, 52% of patients who underwent complete resection) experienced a superb 5-year survival probability of 53.3%, although interestingly there was no significant difference between patients with ypN1 and ypN2 disease (38.5 and 30.8%, respectively).

From a total dose perspective, RTOG 0229 was a multi-institutional prospective study that treated patients with CRT to a total dose of 61.2 Gy with subsequent surgery, essentially a curative dose even without subsequent surgery (11). Out of the 57 initial patients, 56 were eligible for resection and 37 patients ultimately underwent surgery. Most of the patients who did not go to surgery had unresectable or metastatic disease, or were medically inoperable. Forty-three patients had post-RT mediastinal sampling (either at surgery or mediastinoscopy), and 27 patients (63%) experienced mediastinal clearance. The 2-year progression-free and overall survival probabilities were 33 and 54%, respectively. Patients with mediastinal clearance had a 2-year survival probability of 67%, which rose to 75% if they underwent surgical resection. There was only one postoperative death and 14% incidence of grade 3 postoperative pulmonary complications. The survival outcomes for the whole cohort are encouraging, although one cannot discount selection bias for the favorable overall survival results. This result appears to be reproducible, as a small RTOG randomized phase II study using induction CRT (60 Gy) with or without panitumumab—powered to see an improvement in mediastinal clearance—ended up with a similar probability of downstaging (68.2%) in the control arm (12). Yet although this higher dose appears to be to tolerable, the mediastinal CR rate (63–68%) is not so much greater than the comparable rate from SWOG 8805, which used 45 Gy.

Indeed, one must remember that favorable biology is a potent confounder of the relationship between mediastinal clearance and survival. Patients with responsive disease will have improved survival no matter how they are treated, as well as improved mediastinal sterilization rates: aiming to improve mediastinal downstaging with intensified local therapy in this population will only translate into a marginal, if any, improvement in survival.

## Intergroup 0139

Uncertainty about the utility of surgical resection after CRT led to the critical Intergroup 0139 trial, which compared the induction paradigm of SWOG 8805 with definitive CRT (61 Gy) for approximately 400 patients with pN2 stage IIIA NSCLC (13). Both arms received concurrent etoposide and cisplatin. Although the study was designed to answer whether trimodality therapy is superior, the results have been used to support treatment with either treatment approach. With a median follow-up of 69.3 months for surviving patients, there was no significant difference in overall survival [hazard ratio (HR) 0.87, *p* = 0.24, with the 5-year survival probabilities of 27 versus 20% favoring surgery]. Progression-free survival was significantly better for patients in the surgery arm, doubling from 11 to 22% at 5 years. The patterns-of-failure analysis suggested that primary tumor control was the sole oncologic benefit from resection, as it significantly reduced the local-only relapses (22 vs. 10%).

One of the salient findings from the trial, though, revolved around treatment-related mortality, as 14 patients (out of 54, 26%) died after pneumonectomy, most of whom (*n* = 11) had a right-sided pneumonectomy, resulting in a mortality rate of 40% in this subset. This result prompted the authors to perform an unplanned subset analysis, matching patients who underwent a lobectomy with patients in the definitive CRT arm, and similarly matching individuals who underwent a pneumonectomy with patients in the CRT arm. As expected, among patients in the lobectomy comparison, surgery was associated with significantly improved overall survival (36 vs. 18% at 5 years, *p* = 0.002), whereas there was no significant difference in the pneumonectomy comparison. This result has led to the problematic and flawed interpretation that if patients are able to undergo a lobectomy (or if they are converted to a lobectomy with induction treatment), then they will gain a survival benefit from the resection.

The issue with this conclusion is that patients were not stratified by proposed surgery, and thus not only unknown confounders could have biased this comparison, but also obviously known confounders would prevent a legitimate comparison. The included surgical patients did well by virtue of their receipt of surgery after induction, and potentially very well as shown by the ability to undergo a lobectomy rather than a more involved operation. Indeed, only 71% of analyzed surgical patients underwent a complete resection, so by definition patients in the completely resected lobectomy “cohort” were more favorable than the comparison RT patients, in which there was no post-treatment selection. The comparison was the proverbial apples-to-oranges analysis, although unfortunately a popular conclusion from the paper is that patients who may undergo a lobectomy should be treated with trimodality therapy. Nevertheless, a safer and more statistically grounded assessment is that trimodality therapy improved progression-free survival in comparison to definitive CRT, a result that preserved its place as a potentially viable treatment approach for patients expected to tolerate the aggressive therapy.

## Espatue

While the Intergroup study provided motivation for continuing to explore trimodality therapy, the unexpected post-surgical mortality risk significantly dampened enthusiasm for the approach. There is a second multi-institutional randomized study of definitive CRT versus trimodality therapy that provides additional information on these two treatments (14). In this German study, patients with IIIA (N2) and selected IIIB NSCLC were all given three cycles of induction chemotherapy with cisplatin and paclitaxel, and non-progressors were all treated with hyperfractionated CRT (45 Gy in 30 twice-daily fractions). Patients were re-assessed for operability during the last week of RT, and those eligible for surgery were randomized between completing RT (additional 20–26 Gy in daily fractions) and surgical resection.

Although the study was closed early, 246 patients were enrolled, and after the serial treatments 161 patients were randomized. Seventy (out of 81) of the surgical patients went to resection, of whom 66 had an R0 resection. A total of 5 (7%) patients experienced a grade 5 toxicity after surgery, but only one death was following pneumonectomy. After a median follow-up of 78 months, there were no differences in progression-free (35 vs. 32% favoring CRT) or overall (40 vs. 44% favoring surgery) survival. Unfortunately, the patterns-of-failure were not reported.

This trial differs from the Intergroup study in several ways. First and perhaps most important, patients were selected for response (or progression) prior to randomization. Thus, the cohort who made it to randomization were responding to treatment, so perhaps they were more likely to respond to RT as well. Second, the vast majority of patients underwent pre-treatment PET staging, so individuals with previously occult metastatic disease were not included in the study, increasing the likelihood of seeing a survival advantage with improved local therapy. And yet, there was no difference in overall survival.

What can we conclude from these two phase III studies? One straightforward answer is that there is no obvious winner, but for patients who may not tolerate anatomic surgical resection—a non-trivial if not large percentage of the population—definitive CRT is the obvious treatment of choice. On the other hand, the Intergroup study suggests that without first selecting patients with induction therapy, progression-free survival is improved following surgical resection *via* improved local/primary control. Thus, for high performing patients who are at greatest risk for local first progression, trimodality therapy may be reasonable.

## Comparing Induction Chemotherapy with Induction CRT

There is a long history of trials comparing induction chemotherapy followed by surgery with surgery alone, with the majority of those trials showing an overall survival advantage with neoadjuvant systemic treatment (15). Two phase III randomized trials have, thus, asked the natural question of whether preoperative CRT provides any additional benefit to preoperative chemotherapy alone. In the first study, the German Lung Cancer Cooperative Group treated over 500 patients with induction chemotherapy, with non-progressors then randomized between preoperative hyperfractionated CRT (45 Gy in 3 weeks) followed by surgery, or immediate surgery, with postoperative RT (54–68 Gy) (16). Out of the original 279 patients assigned to CRT, 231 finished induction chemotherapy, 208 started CRT, and 142 patients underwent surgery (54% of original cohort). A total of 279 patients were assigned induction chemotherapy alone, of whom 230 patients finished chemotherapy, and 154 patients underwent surgery (59% of original cohort). From a toxicity perspective, patients receiving CRT experienced significantly increased grade 3 or higher hematologic toxicity (10 vs. 1%) and esophagitis (19 vs. 4%), but less pneumonitis (1 vs. 7%). There were no significant differences in surgical mortality, although numerical trends favored preoperative chemotherapy alone (9 vs. 5%) overall surgical mortality, with mortality after pneumonectomy doubled (14 vs. 6%).

Essentially every surrogate endpoint favored preoperative CRT, with more patients undergoing complete resection (75 vs. 60%, *p* = 0.0008), nodal downstaging to N0-1 (46 vs. 29%, *p* = 0.02), and histopathologic response greater than 90% (60 vs. 20%, *p* < 0.0001). As expected, patients undergoing a complete resection experienced superior survival, as did individuals with mediastinal downstaging. Despite these results, though there were no differences in progression-free or overall survival, or in the patterns-of-failure.

An important question is why such clear pathologic differences did not translate into improved overall survival with CRT. One possible explanation is simply that the superior responses in CRT are due to the increased time between the start of induction therapy and pathologic evaluation, and the chemotherapy cohort would have had an increased pCR rate if more time had transpired. Another relevant hypothesis is that pathologic response largely reflects micrometastatic sensitivity to chemotherapy. Although radiation therapy increases the local pathologic response by adding an additional cytotoxic therapy, the prognostic information is largely held in the chemotherapy response, which is obviously unchanged given that both arms received the same systemically active chemotherapy. Since any chemoresistant disease is ultimately removed by surgery, and then followed by radiation therapy, there would be no expected locoregional control differences in the two arms. These two explanations are important considerations as one tries to interpret the strengths and weaknesses of the two treatment paradigms.

The second trial was smaller cooperative group study perform by SAKK (Swiss Group for Clinical Cancer Research), and the results generally echoed the German study (17). In this study, operable stage IIIA/N2 patients were treated with three cycles of induction cisplatin and docetaxel, and non-progressors received either underwent immediate surgery or RT alone (44 Gy in 22 daily fractions) followed by surgery. An additional difference between these two trials is that postoperative RT was only delivered for an R1 or R2 resection (16% of patients in total). Although this study benefited from utilized a third-generation induction doublet, toxicity from induction chemotherapy was high—45% of patients in the RT arm and 60% in the chemotherapy arm developed a grade 3 or 4 toxic effect. In part likely due to the absence of concurrent chemotherapy, toxicity with RT was mild, with only 9 total grade 3 events. The addition of preoperative RT did not increase the risk of postoperative complications or mortality, the latter of which was quite low (3%) and only seen in the chemotherapy-alone patients.

Patients treated with trimodality therapy were more likely to have an objective response (61 vs. 44%), but that was the only statistical difference between the arms. There were clear numerical benefits in resection score and nodal downstaging (e.g., mediastinal clearance in 64 versus 53% of patients), but no comparisons were statistically significant. There were no statistically significant differences in event-free survival, overall survival, or patterns-of-failure, although the latter were not clearly specified. Overall survival outcomes were favorable, with median overall survival times of 37.1 and 26.2 months for induction chemotherapy and radiation and chemotherapy alone, respectively, with 5-year overall survival of approximately 40%.

It is important to remember, though, that patients were operable and generally had low-bulk disease. Moreover, what the authors term the “chemoradiation” arm was actually sequential therapy and is far removed from conventional preoperative combined modality therapy. Since it has been long established that radiation alone is an unimpactful neoadjuvant strategy (18), it is difficult to translate these results into routine practice. The study was also underpowered to compare these two treatments in a relatively favorable patient population, with just over 100 patients per arm: expecting a 50% increase in median survival with the addition of preoperative radiation therapy alone is not a reasonable assumption.

## Determining the Optimal Neoadjuvant Approach

In order to determine the optimal treatment paradigm for a given patient, one must first recognize the unclear benefits of adding surgical resection to stage III NSCLC. Two large phase III trials have failed to show a consistent oncologic benefit to resection over CRT alone, and postoperative morbidity—before even considering mortality—is not trivial and potentially quite life-altering for patients. Patients in whom there is any legitimate question of surgical fitness should not be considered for bi- or trimodality therapy incorporating surgery.

For the relatively small subset of patients who clearly have operable disease and are straightforward operative candidates, the treatment options are more debatable. Certainly definitive CRT is a viable and possibly always the correct approach. Yet the Intergroup study is convincing that tolerable surgical resection reduces the probability of local failure, and there are certainly clinical scenarios in stage III NSCLC in which primary tumor recurrence is the greatest risk for the patient. For example, patients with large primaries and limited mediastinal disease will often fall into this category.

Once the idea of introducing surgery is entertained, which neoadjuvant approach is best? It is clear from the literature that there is no significant overall survival benefit with induction CRT over chemotherapy alone. And while there is often more concern over postoperative morbidity following combined treatment, the recent data from Europe should allay most fears about a meaningful increase in complications, provided there is surgeon and institutional experience in surgery following induction treatment. In addition, if the surgical technique needed to achieve an R0 resection is so complicated that radiation treatment may significant complicate the procedure, then resection probably is not such a good idea!

So the treatment recommendations ultimately hinge on physician and patient preferences. Favoring chemotherapy alone is the recognition that many patients who ultimately go to resection can be spared any RT, provided there is a complete resection. There is certainly some value in omitting RT. Moreover, novel (or at least more active) chemotherapy agents may be easily delivered without concurrent RT, so patients may benefit from histology-directed agents rather than a regimen that is compatible with radiation treatment. Yet it is completely unclear whether the chosen chemotherapy doublet is that impactful in the non-metastatic setting.

On the other hand, a major risk of preoperative chemotherapy alone is the possibility that surgery becomes infeasible for whatever reason, and then the patient requires definitive CRT for an opportunity for cure. This scenario is not uncommon. In the German randomized study, which did not screen for operability, only 59% of patients ultimately went to surgery. That number was substantially higher in the SAKK trial, which only included operable stage IIIA patients, but even still 10% of patients did not make it to the operative room, and 8% of operated patients had gross residual disease. For those individuals who then need definitive CRT, they will have already received induction chemotherapy, which has been shown not to improve outcomes relative to definitive CRT (7), and their tolerability of treatment will likely be altered due to their recent exposure to systemic therapy.

By contrast, initiating CRT preserves all definitive treatment options without creating the possibility of delivering ultimately fruitless systemic therapy. Such treatment also will clearly increase the pathologic response, but in fairness, as mentioned above, the implications of this improvement relative to chemotherapy alone are still questionable. Although 45 Gy should be considered the standard induction dose based on Intergroup 0139, stopping at 45 Gy and then hoping the surgeon still considers the case operable is always anxiety-producing, because if surgery is not ultimately performed, the patient has received inadequate local therapy.

Instead, regardless of the preoperative likelihood that the patient will go to surgery, my preference is to deliver radical dose CRT to 60 Gy, which has been shown to be tolerable in a multi-institutional setting, and then selectively choose patients for resection. This minimizes the possibility of delivering insufficient local therapy—especially when patients are marginally operable—while providing the opportunity for subsequent surgery in the appropriate situation.

From an academic standpoint, patient scenarios can be divided into four groups based on tumor and nodal response. Patients who theoretically have a complete primary and nodal response do not need surgery, as the marginal gain in local control will be outweighed by toxicity. Patients with progressive or persistent primary and nodal disease do not need surgery, as the prognosis is too poor to warrant the risks of resection. Patients with persistent mediastinal disease but a complete primary response do not need surgery, as the risk of metastasis outweighs the very small improvement in local control. Finally, patients with a mediastinal response but persistent local disease may very well gain from resection, as micrometastastic disease may have been sterilized by chemotherapy but the local treatment has not fully responded. It is this latter cohort, defined by imaging and ideally mediastinal evaluation, for whom the therapeutic ratio favors trimodality therapy. Unfortunately, patients cannot be easily placed into one of these “boxes,” as restaging modalities are insufficiently accurate to determine local and nodal response (19, 20), but this basic paradigm roughly guides how we can think about intensified local therapy in this disease.

## The Future

One can divide future progress on this question to be divided into evolutionary versus revolutionary innovations. With time, more genomic and radiomic predictors of locoregional and distant control may be developed, providing either pre-treatment or mid-treatment information on the expected outcomes. Such prognostic information could provide valuable non-invasive information on the likelihood of the clearing the mediastinum or obtaining primary tumor control prior to deciding on surgery. Such technology would be a welcome innovation but would likely not meaningfully raise the proverbial tail of the survival curve, which has largely plateaued. A more revolutionary step would be the introduction of novel systemic therapies that more effectively control micrometastatic disease, raising the impact of improved locoregional control. Of course, such chemotherapy may also reduce local progression, minimizing the benefit of surgical resection. For example, there was a recent announcement that a phase III randomized trial of adjuvant durvalumab, an immunotherapy drug that blocks PD-L1 (programmed death-ligand 1), improved progression-free survival in stage III patients treated with definitive CRT (21). The future integration of surgical resection into stage III NSCLC may grow or shrink, depending on how these exciting therapies influence the disease course.

## Conclusion

Although it is debatable whether surgical resection plays any role in stage III NSCLC, if one pursues a preoperative paradigm, either induction CRT or chemotherapy alone are viable treatment approaches. The strengths and weaknesses of both approaches have been detailed above, and from a practical, “real-world” perspective, a strong argument has been made to favor the incorporation of RT into the neoadjuvant program. Regardless of the final treatment, however, central to treatment success is close coordination between medical, radiation, and surgical oncologists. Collaboration and open dialog are critical to ensure the safest and most efficacious treatment in this challenging patient population.

## Author Contributions

The author confirms being the sole contributor of this work and approved it for publication.

## Conflict of Interest Statement

The author declares that the research was conducted in the absence of any commercial or financial relationships that could be construed as a potential conflict of interest.
